# Standardized Ileal Amino Acid Digestibilities of Sorghums from Different Sources in Yellow-Feathered Chickens and Their Prediction Models

**DOI:** 10.3390/ani16111747

**Published:** 2026-06-05

**Authors:** Xiaoyan Cui, Yucai Liu, Wenpeng Chen, Qianwen Yuan, Liyang Zhang, Shengchen Wang, Tingting Li, Yun Hu, Xugang Luo

**Affiliations:** 1 Poultry Mineral Nutrition Laboratory, College of Animal Science and Technology, Yangzhou University, Yangzhou 225009, China; 2 Mineral Nutrition Research Division, State Key Laboratory of Animal Nutrition and Feeding, Institute of Animal Science, Chinese Academy of Agricultural Sciences, Beijing 100193, China

**Keywords:** sorghum, chemical composition, medium-growing yellow-feathered chicken, standardized ileal amino acid digestibility, prediction model

## Abstract

Prediction models for standardized ileal amino acid digestibilities (SIAADs) of sorghums in yellow-feathered chickens have not been previously reported. This study characterized the chemical composition of 10 sorghum samples from different sources, assessed their SIAADs in medium-growing yellow-feathered chickens, and subsequently developed and validated prediction models based on chemical composition and amino acid profiles. A total of 276 Tianluma roosters (60 d of age) were randomly assigned by body weight (average 1.32 kg per bird) to 11 dietary treatments, including a nitrogen-free diet (NFD) group and 10 sorghum-based diet groups. Each treatment included 6 replicate cages, with 4 birds per replicate cage for the sorghum-based diet groups and 6 birds per replicate cage for the NFD. Birds were fed the experimental diets from d 63 to 67, after which ileal digesta were collected to determine SIAADs of 10 sorghum samples. Data from 9 sorghum samples were used to establish prediction equations using stepwise regression, while the remaining sample was used for model validation. Sorghum source significantly influenced (*p* ≤ 0.002) the SIAADs of most amino acids. Arginine (Arg) exhibited the highest standardized ileal digestibility (SID) (68.2%), whereas tyrosine (Tyr) showed the lowest value (49.0%). Eighteen preliminary prediction models were developed for the SIDs of valine (Val, *R*^2^ = 0.981, *p* = 0.001), methionine (Met, *R*^2^ = 0.978, *p* < 0.001), isoleucine (Ile, *R*^2^ = 0.983, *p* < 0.001), leucine (Leu, *R*^2^ = 0.981, *p* < 0.001), threonine (Thr, *R*^2^ = 0.748, *p* = 0.016), phenylalanine (Phe, *R*^2^ = 0.981, *p* < 0.001), lysine (Lys, *R*^2^ = 0.988, *p* < 0.001), histidine (His, *R*^2^ = 0.988, *p* = 0.004), Arg (*R*^2^ = 0.986, *p* < 0.001), tryptophan (Trp, *R*^2^ = 0.934, *p* < 0.001), aspartic acid (Asp, *R*^2^ = 0.986, *p* < 0.001), serine (Ser, *R*^2^ = 0.980, *p* < 0.001), glutamic acid (Glu, *R*^2^ = 0.988, *p* < 0.001), glycine (Gly, *R*^2^ = 0.898, *p* = 0.007), alanine (Ala, *R*^2^ = 0.983, *p* < 0.001), cysteine (Cys, *R*^2^ = 0.968, *p* = 0.003), Tyr (*R*^2^ = 0.898, *p* = 0.001), and proline (Pro, *R*^2^ = 0.944, *p* = 0.002). The models for the SIDs of Lys, His, and Glu exhibited the highest coefficients of determination (*R*^2^ = 0.988, *p* ≤ 0.004), whereas the model for the Thr SID exhibited the lowest fit (*R*^2^ = 0.748, *p* = 0.016). Except for Leu and Tyr, the predicted values of the remaining amino acids were generally consistent with the determined values in the validation sample. These preliminary models provide a basis for estimating amino acid digestibilities in sorghums for medium-growing yellow-feathered chickens.

## Simple Summary

This study evaluated the standardized ileal amino acid digestibility (SIAAD) of sorghum from different sources in medium-growing yellow-feathered chickens and developed prediction models based on chemical composition and amino acid profiles. Results showed that the sorghum source significantly affected amino acid digestibility, with tannins negatively influencing nutrient utilization. Most preliminary models showed promising predictive performance. These findings improve our understanding of factors affecting amino acid digestibility in sorghum and provide a basis for the future development of prediction tools for precision feed formulation in poultry production.

## 1. Introduction

Sorghum is widely used in poultry feeding owing to its extensive availability, strong drought tolerance, and comparatively high levels of metabolizable energy and starch [1]. Although modern sorghum cultivars have undergone substantial genetic improvement, considerable variation still exists in their nutritional value and amino acid utilization in chickens [2,3]. This variation arises from differences among cultivars as well as antinutritional compounds, including tannins and phytate, which can interact with nutrients or digestive enzymes and subsequently decrease protein and amino acid digestibility [4,5,6]. Furthermore, sorghum composition, including crude protein (CP), starch, and non-starch polysaccharides, can be markedly affected by genotype, growing environment, geographic origin, and processing methods [7,8,9]. These factors collectively shape the nutritional quality and digestibility of sorghum-based diets. Consequently, precisely evaluating amino acid digestibility in sorghum is critical for optimizing feed formulation, maximizing nutrient utilization, reducing nitrogen excretion, and mitigating feed resource shortages. This is particularly important for medium-growing yellow-feathered chickens, a major broiler type in China that exhibits slower growth compared with fast-growing white-feathered broilers.

Standardized ileal amino acid digestibility (SIAAD) has been widely recommended as the most reliable metric for assessing amino acid availability in feed ingredients, as it corrects for basal endogenous losses (BELs) and provides additive values necessary for precise diet formulation [10,11]. Previous studies have successfully applied SIAAD to characterize amino acid digestibility in various cereals and protein meals and have also demonstrated the feasibility of developing predictive models based on chemical composition to quickly predict feed amino acid digestibility in poultry [12,13,14]. However, SIAAD data for sorghum in broilers remain scarce, and no study has reported prediction equations for the SIAADs of sorghum in yellow-feathered chickens.

Therefore, we hypothesized that the SIAAD values of sorghums for medium-growing yellow-feathered chickens could be accurately predicted from their chemical composition and amino acid profiles. To examine this hypothesis, in the present study, (1) the chemical composition and anti-nutritional factors of 10 sorghum samples were analyzed; (2) their SIAAD values in chickens were assessed; and (3) prediction models for SIAADs based on chemical composition and amino acid profiles were developed, and the accuracies of these models were validated.

## 2. Materials and Methods

### 2.1. Animal Ethics

All experimental procedures involving animals were conducted in accordance with the guidelines of the Animal Use Committee of the Ministry of Agriculture of China (Beijing, China). The experimental protocol was reviewed and approved by the Ethics Committee of the Department of Animal Science and Technology, Yangzhou University (permit number: SYXK (Su) 2021-0027).

### 2.2. Experimental Design and Treatments

The experiment was conducted using a completely randomized design. Eleven dietary treatments were established, consisting of 1 nitrogen-free diet (NFD) group and 10 sorghum-based diet groups.

### 2.3. Sorghums, Animals, and Diets

Ten sorghum samples (designated A1 to A10) were collected from suppliers located in different regions. Basic information regarding the origins of the 10 sorghum samples is provided in Table 1.

A total of 300 Tianluma roosters (55 d of age) were obtained from Huai’an Wen’s Livestock Co., Ltd. (Huai’an, China) and reared in stainless-steel cages equipped with separate feeders and drinkers. Tianluma roosters were used as an experimental model representing medium-growing yellow-feathered chickens. Throughout the experimental period, ambient temperature, relative humidity, and lighting conditions were maintained in accordance with standard management recommendations for yellow-feathered broilers. On d 60, birds were fasted and weighed to confirm that body weights were consistent with reference standards for yellow-feathered chickens of the same age. Afterward, 276 birds with similar body weights (average 1.32 kg per bird) were randomly allocated to 11 dietary treatments. Each treatment included 6 replicate cages, with 4 birds per replicate cage for the sorghum-based diet groups and 6 birds per replicate cage for the NFD. Six birds were allocated to each replicate cage in the NFD treatment to ensure adequate ileal digesta collection because the NFD generally yields lower digesta output. Four birds per replicate cage in sorghum-based diets were sufficient to obtain representative samples for digestibility analysis. All birds were given the same commercial pelleted diet and tap water ad libitum from d 55 to 62, after which the experimental diets in pellet form and tap water were provided ad libitum from d 63 to 67.

The NFD was formulated primarily with corn starch, glucose, cellulose, and a vitamin-mineral premix. The sorghum diets were formulated using sorghum as the sole source of CP and amino acids, supplemented with a vitamin-mineral premix. All experimental diets contained 0.4% titanium dioxide (TiO_2_) as an indigestible marker. Except for amino acids, all diets were prepared to satisfy the nutrient requirements of yellow-feathered broilers at the relevant growth stage according to NY/T 3645-2020 [15]. The chemical composition data for the sorghum samples are presented in Table 2, and the ingredient composition and nutrient levels of the experimental diets are shown in Table 3.

### 2.4. Sample Collections and Preparations

Representative samples of sorghums and experimental diets were collected and ground to pass through a 0.45 mm screen with a stainless-steel mill prior to chemical analyses.

On d 67, all 276 birds were euthanized by carbon dioxide asphyxiation without prior fasting. Ileal digesta were then collected immediately from the posterior half of the ileum, excluding the distal 2 cm, using a gentle squeezing method [16]. After collection, the digesta samples were stored at −20 °C for subsequent processing. The frozen samples were then freeze-dried. Digesta collected from birds within the same replicate cage were pooled, ground thoroughly to ensure homogeneity, and stored at −20 °C for later analyses.

### 2.5. Measurement Indicators and Methods

Moisture, CP, ether extract (EE), crude ash (CA), crude fiber (CF), neutral detergent fiber (NDF), and acid detergent fiber (ADF) contents in sorghum samples or experimental diets were analyzed according to AOAC official methods 934.01, 976.05, 954.02, 942.05, 978.10, 2002.04, and 973.18, respectively [17]. Starch (ST) content in sorghum samples was quantified with a commercial assay kit (Beijing Solebo Technology Co., Ltd., Beijing, China). Phytic acid (PA) concentration in sorghum samples was analyzed with a commercial determination kit (Suzhou Keming Biotechnology Co., Ltd., Suzhou, China). Tannin (TN) content in sorghum samples was measured following the procedure of Luzardo-Ocampo et al. [18]. Amino acid concentrations in sorghums, experimental diets, and ileal digesta were determined according to AOAC official methods [17], including method 994.12 for most amino acids, method 985.28 for methionine (Met) and cysteine (Cys), and method 988.15 for tryptophan (Trp). TiO_2_ concentrations in diets and digesta were determined using the method reported by Titgemeyer et al. [19]. Previous studies have demonstrated that TiO_2_ provides a reliable marker recovery in poultry digestibility experiments [20].

### 2.6. Calculations

The BEL, apparent ileal amino acid digestibility (AIAAD), and SIAAD were calculated on a dry matter basis using the following equations:

BEL (mg/kg) = (W_I_ × W_II_)/W_III_ (1)

AIAAD (%) = 100 − (D_I_ × D_II_)/(D_III_ × D_IV_) × 100 (2)

SIAAD (%) = AIAAD + BEL/D_III_ × 100 (3)

where W_I_ represents the amino acid content (mg/kg) in ileal digesta of birds fed the NFD; W_II_ is the TiO_2_ content (mg/kg) in the NFD; W_III_ is the TiO_2_ content (mg/kg) in ileal digesta of birds fed the NFD; D_I_ is the amino acid content (mg/kg) in ileal digesta of birds fed sorghum-based diets; D_II_ is the TiO_2_ content (mg/kg) in sorghum-based diets; D_III_ is the amino acid concentration (mg/kg) in sorghum-based diets; and D_IV_ is the TiO_2_ content (mg/kg) in ileal digesta of birds fed sorghum-based diets.

### 2.7. Statistical Analyses

AIAAD and SIAAD data were analyzed by one-way ANOVA using the GLM procedure in SAS 9.4. When treatment effects were significant, mean values were separated by the LSD test [21]. Pearson correlation analysis was conducted to evaluate relationships among chemical composition, amino acid profiles, and SIAAD values of sorghums. Prediction models were generated by stepwise regression using data from 9 sorghum samples (A1–A5 and A7–A10), while one sample (A6) was randomly chosen for validation with the RANDGEN function in SAS [22,23]. Each replicate cage was considered the experimental unit, and statistical significance was declared at *p* < 0.05.

## 3. Results

### 3.1. Chemical Compositions of Sorghum Samples

As shown in Table 2, the ranges of moisture, CP, EE, CF, NDF, ADF, CA, nitrogen-free extract (NFE), ST, PA, and TN contents in the 10 different sources of sorghums were 11.32 to 15.09%, 7.24 to 10.21%, 2.02 to 3.97%, 1.30 to 2.94%, 7.03 to 17.49%, 2.42 to 5.10%, 1.33 to 1.72%, 69.89 to 74.80%, 64.38 to 69.71%, 0.69 to 0.95%, and 0.05 to 1.45%, respectively. Among all chemical traits, NFE showed the lowest coefficient of variation (CV; 2.10%), while TN exhibited the greatest variability (CV = 81.77%). For essential amino acids (EAAs), the contents of valine (Val), Met, isoleucine (Ile), leucine (Leu), threonine (Thr), phenylalanine (Phe), lysine (Lys), histidine (His), arginine (Arg), and Trp ranged from 0.44 to 0.57%, 0.06 to 0.12%, 0.31 to 0.41%, 1.06 to 1.44%, 0.28 to 0.36%, 0.43 to 0.58%, 0.17 to 0.27%, 0.18 to 0.26%, 0.66 to 1.12%, and 0.08 to 0.11%, respectively. The CV values of EAAs ranged from 9.10 to 26.98%, with the highest variation observed for Met and the lowest for Val. For non-essential amino acids (NEAAs), the contents of aspartic acid (Asp), serine (Ser), glutamic acid (Glu), glycine (Gly), alanine (Ala), Cys, tyrosine (Tyr), and proline (Pro) ranged from 0.57 to 0.77%, 0.38 to 0.52%, 1.77 to 2.43%, 0.26 to 0.35%, 0.75 to 1.01%, 0.05 to 0.13%, 0.20 to 0.36%, and 0.88 to 1.20%, respectively. The CVs of NEAAs ranged from 9.65 to 35.27%, in which Cys showed the greatest variability, whereas Gly was the most stable.

### 3.2. AIAADs of Sorghums for Chickens

Table 4 shows that the sorghum source influenced (*p* ≤ 0.001) the apparent ileal digestibilities (AIDs) of 17 amino acids in chickens, whereas Lys was not affected. For EAAs, the AID values of Val, Met, Ile, Leu, Thr, Phe, Lys, His, Arg, and Trp ranged from 17.8 to 54.7%, 23.4 to 68.9%, 16.1 to 64.7%, 23.6 to 75.2%, 17.4 to 43.0%, 25.7 to 70.9%, 32.1 to 53.7%, 21.9 to 59.5%, 29.8 to 71.3%, and 24.3 to 62.6%, respectively. Among these, Arg exhibited the greatest mean AID value (53.8%), whereas Thr showed the lowest (30.9%). The CV values for EAAs were 33.2% for Val, 35.9% for Met, 40.7% for Ile, 40.8% for Leu, 25.9% for Thr, 35.8% for Phe, 17.3% for Lys, 31.9% for His, 27.0% for Arg, and 31.7% for Trp. Among the EAAs, Lys exhibited the greatest stability in AID values, whereas Leu showed comparatively higher variability. For NEAAs, the AID values of Asp, Ser, Glu, Gly, Ala, Cys, Tyr, and Pro ranged from 25.3 to 63.3%, 18.0 to 56.4%, 25.3 to 73.6%, 27.8 to 58.6%, 29.1 to 75.5%, 8.5 to 59.0%, 12.5 to 74.2%, and 15.6 to 62.3%, respectively. Ala showed the highest mean AID value (52.3%), whereas Cys exhibited the lowest (32.0%). The CV values for NEAAs were 25.7% (Asp), 32.4% (Ser), 37.4% (Glu), 23.2% (Gly), 35.0% (Ala), 47.8% (Cys), 59.4% (Tyr), and 36.9% (Pro). Gly was the least variable among these amino acids, whereas Tyr showed the greatest variation.

### 3.3. SIAADs of Sorghums for Chickens

As shown in Table 5, sorghum source influenced (*p* ≤ 0.002) the standardized ileal digestibilities (SIDs) of 17 amino acids in chickens, with the exception of Lys. For EAAs, the SID values of Val, Met, Ile, Leu, Thr, Phe, Lys, His, Arg, and Trp ranged from 36.3 to 70.5%, 32.8 to 76.6%, 28.6 to 76.4%, 29.9 to 81.9%, 42.4 to 64.3%, 35.6 to 81.2%, 54.6 to 75.1%, 33.0 to 67.7%, 47.1 to 83.7%, and 40.1 to 75.8%, respectively. Among these amino acids, Arg exhibited the greatest mean SID value (68.2%), whereas His had the lowest (51.8%). The CV values of EAAs were 21.5% (Val), 29.8% (Met), 30.5% (Ile), 35.4% (Leu), 13.8% (Thr), 28.7% (Phe), 11.0% (Lys), 25.1% (His), 19.5% (Arg), and 22.7% (Trp). Lys displayed the greatest stability in SID values, whereas Leu exhibited comparatively higher variability. For NEAAs, the SID values of Asp, Ser, Glu, Gly, Ala, Cys, Tyr, and Pro ranged from 43.1 to 76.8%, 40.3 to 74.0%, 34.1 to 81.0%, 44.5 to 72.5%, 35.4 to 82.0%, 29.5 to 73.6%, 19.1 to 80.7%, and 26.2 to 72.0%, respectively. Among these, Asp exhibited the highest mean SID value (61.8%), whereas Tyr showed the lowest (49.0%). The CV values of NEAAs were 18.4% (Asp), 20.5% (Ser), 31.7% (Glu), 15.6% (Gly), 30.7% (Ala), 26.3% (Cys), 49.3% (Tyr), and 30.7% (Pro). Gly was the least variable, while Tyr varied the most.

### 3.4. Correlations Among Chemical Compositions, Amino Acid Profiles, and SIAADs of Sorghums for Chickens

Table 6 shows that TN content in sorghum was negatively correlated (*p* < 0.05) with the SID values of 16 amino acids, except for Lys and Cys. The results presented in Table 7 indicate a positive correlation (*p* < 0.05) between Leu content and the SID of Glu. Table 8 shows that Tyr content was positively related (*p* < 0.05) to the SIDs of Thr and Lys, while Pro content was positively related (*p* < 0.05) to the SIDs of Met, Leu, Phe, Trp, Asp, Ser, Glu, and Ala. No other significant correlations were detected.

### 3.5. Prediction Models for SIAADs of Sorghums in Chickens

The prediction models developed for SIAADs are summarized in Table 9. In total, 18 preliminary prediction equations were developed for chickens, with coefficients of determination (*R*^2^) ranging from 0.748 to 0.988 (*p* < 0.02). The primary predictors included in the equations were CA, TN, Met, and Trp. The equations for Lys, His, and Glu SIDs exhibited the highest coefficients of determination, with *R*^2^ values of 0.988 (*p* ≤ 0.004), whereas the Thr SID equation had the lowest fit (*R*^2^ = 0.748, *p* = 0.016). Importantly, the models for Val, Met, Ile, Leu, Phe, Lys, His, Arg, Trp, Asp, Ser, Glu, Ala, Cys, and Pro all yielded *R*^2^ values greater than 0.90, indicating a good fit to the current dataset.

### 3.6. Validation of the SIAAD Prediction Models for Sorghum in Chickens

To provide a preliminary evaluation of the developed models, 1 sorghum sample (A6) was randomly chosen for validation. Table 10 presents the predicted SID values for individual amino acids in sorghum A6. The differences between determined and predicted SID values were 0.3% (Val), 1.0% (Met), 1.3% (Ile), 13.1% (Leu), 0.0% (Thr), 2.1% (Phe), 2.0% (Lys), 0.0% (His), 2.6% (Arg), 1.4% (Trp), 0.2% (Asp), 2.1% (Ser), 1.3% (Glu), 4.7% (Gly), 1.2% (Ala), 15.3% (Cys), 15.0% (Tyr), and 1.6% (Pro). With the exception of Leu and Tyr, the predicted SID values for all other amino acids fell within the determined mean ± standard deviation (SD) ranges.

## 4. Discussion

In this study, sorghum samples from different sources were analyzed for chemical composition, and the SIDs of 18 amino acids in medium-growing yellow-feathered chickens were evaluated, after which SIAAD prediction models were established and validated. The results demonstrated that variations in sorghum chemical compositions (CA, TN, PA, EE, NDF, and ST) and amino acid profiles (Ser, Met, Trp, Gly, Tyr, Ala, and Arg) could be used to estimate the digestibilities of most amino acids. Except for Leu and Tyr, predicted SID values generally agreed with the determined values in the validation sample. These results support our hypothesis and provide novel data specific to medium-growing yellow-feathered chickens for the application of chemical composition-based prediction models for precision amino acid nutrition.

The chemical composition of sorghum can vary considerably depending on cultivar, environmental conditions during growth, and post-harvest handling practices [4,7,8]. Feng et al. [11] evaluated 10 sorghum samples collected from 9 cities in China, and the concentrations of CF, NDF, ADF, CP, CA, EE, ST, TN, and amino acids they reported were broadly consistent with the values obtained in the present study. In contrast, Sasia et al. [3] examined 8 sorghum samples without tannin from the United States and found somewhat higher CP contents but lower NDF levels compared with those observed here, which may be attributable to differences in sorghum origin. The present study exhibited relatively low variability (CVs < 10%) in moisture, ST, NFE, CA, Phe, Val, Thr, Ile, Ser, and Gly contents among the 10 sorghum samples. Conversely, higher variation (CVs > 10%) was observed for CP (10.06%), EE (21.13%), CF (25.99%), NDF (36.45%), ADF (29.64%), PA (10.41%), TN (81.77%), Met (26.98%), Leu (10.44%), Lys (12.88%), His (13.24%), Arg (17.82%), Trp (10.97%), Asp (10.05%), Glu (10.59%), Ala (10.03%), Cys (35.27%), Tyr (17.52%), and Pro (11.34%). The CV values of most chemical components and amino acids observed in the present study were generally consistent with values reported in prior investigations of sorghums sourced from different regions of China [11]. However, the relatively wide variability observed for TN and several amino acids, particularly Met, Cys, and Tyr, highlights the heterogeneity of sorghums as a feed ingredient. Such variability is a prerequisite for the development of robust prediction models, as it allows meaningful associations between composition and digestibility to be identified.

The omission of BELs in AIAAD values may reduce the accuracy of amino acid digestibility estimates [24,25]. In the current study, considerable variation was found in the AIAADs of sorghums, with values ranging from 23.6 to 75.2% for the AID of Leu, 16.1 to 64.7% for Ile, 12.5 to 74.2% for Tyr, and 8.5 to 59.0% for Cys. Such large variations are likely attributable to the susceptibility of AID values to BEL influence. In comparison, SIAAD values more accurately reflect the true availability of amino acids in feed ingredients because BELs are taken into account. Previous studies evaluating AIAADs and SIAADs of sorghums have focused primarily on pigs. Thomas et al. [26] assessed AIAADs in high-lysine, red, and white sorghums for nursery pigs and reported higher digestibility values for most amino acids than those observed in the present study. Similarly, Feng et al. [11] found that the AIAAD values of sorghums for growing-finishing pigs were comparable to those obtained in the present study for Thr, Gly, Ser, Ala, Asp, Tyr, and Pro, but were higher for Ile, Val, Phe, Met, Leu, Glu, Cys, His, and Trp, and slightly lower for Lys and Arg. These differences are likely attributable to species-related variations between pigs and chickens. Because SIAADs are adjusted for BELs, they provide a more accurate reflection of amino acid utilization and are therefore essential for precise dietary amino acid formulation to reduce feed costs and nitrogen losses [10]. In the current study, the sorghum source affected the SID values of 17 amino acids, with the exception of Lys, in chickens. Feng et al. [11] assessed SIAADs in 10 sorghum samples for growing-finishing pigs and reported higher digestibility values for most amino acids than those observed in the present study, whereas Lys and Arg SID values were similar between the two studies. In addition, Sasia et al. [3] reported higher SIAAD values for 18 amino acids in 8 tannin-free sorghum samples fed to white-feathered broilers. These discrepancies may be associated with differences in animal species, sorghum origin, and the presence of TN across studies. Moreover, the SIAAD values observed in the present study were generally lower than those reported in several studies involving fast-growing white-feathered broilers [3,10,27,28]. Although direct comparisons among studies should be interpreted cautiously because of differences in experimental conditions and sorghum sources, these findings may suggest potential genotype-related differences in amino acid utilization. Medium-growing yellow-feathered chickens differ from fast-growing broilers in growth rate, feed intake patterns, gastrointestinal development, and nutrient partitioning, which may contribute to variations in digestive efficiency. However, further studies using the same feed ingredients and experimental conditions are required to confirm whether genotype is a major factor influencing sorghum amino acid digestibility.

In the present study, TN content was negatively correlated with the SIDs of Ile, Val, Thr, Met, Leu, Arg, Phe, Asp, His, Glu, Trp, Ser, Pro, Gly, Tyr, and Ala, whereas positive relationships were identified between selected SIAADs and the contents of Tyr, Leu, and Pro in sorghums for chickens. Similarly, Feng et al. [11] reported that TN was negatively correlated with the SIDs of Lys and Trp in sorghum samples used for growing-finishing pigs. Pan et al. [29] also observed negative correlations between TN and the SIDs of His, Lys, Arg, Val, and Thr in sorghums for growing pigs, further demonstrating the inhibitory effect of TN on amino acid digestibilities. To date, correlations between TN and SIAADs, as well as relationships between SIAADs and amino acid profiles observed in the present study, have rarely been reported for sorghums. Collectively, these findings indicate that TN, as an antinutritional factor, is an important determinant of amino acid digestibility in sorghums fed to poultry. Condensed TN may reduce amino acid digestibility by forming complexes with dietary proteins and digestive enzymes, thereby decreasing protein solubility and enzymatic hydrolysis in the gastrointestinal tract [5]. In addition, TN preferentially interacts with hydrophobic and aromatic amino acid residues [30], which may partly explain the strong negative correlations between TN and multiple SIDs observed in the present study.

The establishment of SIAAD prediction models for chickens provides an important basis for the rapid evaluation of sorghum quality and the precise use of amino acid supplementation. In the current study, 18 prediction models were effectively developed using sorghum chemical composition parameters (CA, EE, TN, PA, NDF, and ST) together with amino acid profiles (Gly, Ser, Ala, Met, Trp, Tyr, and Arg), and the corresponding *R*^2^ values ranged from 0.748 to 0.988. Among these, the models predicting the SIDs of Val, Met, Ile, Leu, Phe, Lys, His, Arg, Trp, Asp, Ser, Glu, Ala, Cys, and Pro all exhibited relatively high *R*^2^ values (>0.90). However, given the limited number of sorghum samples used for model development, these equations should be considered preliminary and require further validation using larger and more diverse datasets. To date, most digestibility prediction models have been developed for pigs. For example, Feng et al. [11] developed prediction equations for growing-finishing pigs and identified TN and Trp as negative predictors, which agree with the findings of the current study. Taken together, these findings highlight the inhibitory role of antinutritional factors, particularly TN, in amino acid digestibility.

One sorghum sample (A6) was randomly chosen to assess the predictive performance of the developed SIAAD models. With the exception of Leu and Tyr, the predicted SID values for Ile, Val, Met, Lys, Thr, Phe, Arg, His, Trp, Gly, Asp, Ala, Ser, Cys, Glu, and Pro were generally consistent with the determined values. These results provide preliminary support for the predictive potential of most models; however, because validation was conducted using only one independent sorghum sample, the robustness and generalizability of the models remain to be further verified. The relatively lower predictive accuracies observed for Leu and Tyr may be associated with their greater biological variability among sorghum samples and their sensitivity to tannin-protein interactions [3]. Because Leu is hydrophobic and Tyr contains aromatic side chains, both amino acids may exhibit stronger interactions with condensed TN, thereby increasing variability in digestibility estimates and reducing prediction accuracy [30]. However, because only one sorghum sample was used for validation, additional studies involving a more diverse set of sorghum samples are required before these models can be applied more broadly in medium-growing yellow-feathered chicken production.

## 5. Conclusions

Prediction models were effectively developed for the SIDs of Val, Ile, Thr, Met, Lys, Phe, His, Arg, Trp, Glu, Asp, Gly, Ser, Cys, Ala, and Pro in sorghums fed to medium-growing yellow-feathered chickens. With the exception of the models developed for Leu and Tyr, which exhibited comparatively lower prediction accuracies, the other models showed promising predictive performance under the conditions of the present study. Nevertheless, additional validation using larger and more diverse sorghum datasets is required before broader application. Accordingly, these models may serve as a useful reference for the rapid prediction of sorghum SIAADs from chemical composition parameters (EE, CA, TN, PA, NDF, and ST) and amino acid profiles (Ser, Met, Trp, Gly, Tyr, Ala, and Arg). In particular, the models predicting the SIDs of Val, Ile, Met, Leu, His, Lys, Phe, Arg, Ser, Trp, Asp, Glu, Cys, Ala, and Pro all produced *R*^2^ values above 0.90, further supporting their predictive reliability. The developed equations should be regarded as preliminary prediction models until their robustness and applicability are confirmed in independent datasets.

## Figures and Tables

**Table 1:** Sources of the 10 tested sorghums.

Number	Sources	Number	Sources
A1	Argentina	A6	Neimenggu
A2	America	A7	Liaoning
A3	Liaoning	A8	Hebei
A4	Jilin	A9	Jilin
A5	Neimenggu	A10	Jiangsu

**Table 2:** Analyzed chemical compositions of the 10 tested sorghum samples (air-dry basis) ^1^.

Item (%)	Sorghum Sources										Mean	SD	CV (%)
	A1	A2	A3	A4	A5	A6	A7	A8	A9	A10			
Moisture	12.47	11.66	15.09	13.40	12.41	11.45	12.35	13.08	11.32	12.54	12.58	1.11	8.80
CP	7.24	9.00	7.85	8.19	8.62	8.77	9.00	9.02	9.88	10.21	8.78	0.88	10.06
EE	2.84	2.98	2.92	3.93	3.97	2.02	3.84	2.43	3.62	3.67	3.22	0.68	21.13
CF	2.46	2.41	2.32	1.30	2.94	1.45	1.57	1.65	2.12	1.97	2.02	0.52	25.99
NDF	9.03	7.67	7.55	7.77	10.89	7.03	9.21	12.15	17.49	16.99	10.58	3.86	36.45
ADF	3.63	2.74	3.16	2.54	2.42	2.47	3.21	4.32	5.02	5.10	3.46	1.03	29.64
CA	1.44	1.33	1.54	1.37	1.46	1.51	1.65	1.55	1.60	1.72	1.52	0.12	8.02
NFE	73.55	72.62	70.28	71.81	70.60	74.80	71.59	72.27	71.46	69.89	71.89	1.51	2.10
ST	65.52	68.33	65.81	64.38	68.45	69.71	68.62	67.59	67.58	66.73	67.27	1.64	2.44
PA	0.71	0.83	0.86	0.69	0.85	0.86	0.95	0.83	0.90	0.73	0.82	0.09	10.41
TN	0.93	0.08	1.05	1.36	1.39	0.05	1.45	0.78	0.06	0.13	0.73	0.60	81.77
EAAs													
Val	0.45	0.57	0.44	0.48	0.48	0.52	0.56	0.54	0.55	0.50	0.51	0.05	9.10
Met	0.06	0.07	0.12	0.06	0.06	0.07	0.07	0.07	0.07	0.07	0.07	0.02	26.98
Ile	0.32	0.41	0.31	0.34	0.34	0.37	0.39	0.39	0.39	0.36	0.36	0.03	9.46
Leu	1.06	1.44	1.06	1.14	1.16	1.32	1.31	1.32	1.35	1.29	1.24	0.13	10.44
Thr	0.28	0.36	0.28	0.30	0.31	0.32	0.36	0.35	0.34	0.31	0.32	0.03	9.76
Phe	0.43	0.58	0.44	0.47	0.48	0.52	0.54	0.56	0.54	0.51	0.51	0.05	9.81
Lys	0.17	0.24	0.21	0.21	0.21	0.21	0.27	0.25	0.23	0.21	0.22	0.03	12.88
His	0.18	0.26	0.18	0.22	0.25	0.22	0.23	0.24	0.25	0.20	0.22	0.03	13.24
Arg	0.66	0.73	0.74	0.68	0.72	0.76	0.84	0.89	1.12	0.96	0.81	0.14	17.82
Trp	0.11	0.10	0.08	0.08	0.08	0.09	0.10	0.10	0.10	0.10	0.09	0.01	10.97
NEAAs													
Asp	0.57	0.75	0.58	0.64	0.64	0.67	0.77	0.72	0.69	0.64	0.66	0.07	10.05
Ser	0.38	0.52	0.39	0.40	0.42	0.45	0.46	0.47	0.44	0.42	0.43	0.04	9.93
Glu	1.77	2.43	1.80	1.91	1.99	2.23	2.24	2.26	2.26	2.16	2.11	0.22	10.59
Gly	0.28	0.35	0.26	0.28	0.28	0.29	0.34	0.31	0.32	0.28	0.30	0.03	9.65
Ala	0.75	1.01	0.75	0.82	0.82	0.93	0.94	0.90	0.94	0.89	0.88	0.09	10.03
Cys	0.05	0.07	0.13	0.05	0.06	0.07	0.06	0.06	0.08	0.07	0.07	0.02	35.27
Tyr	0.29	0.35	0.20	0.31	0.31	0.26	0.27	0.28	0.36	0.22	0.29	0.05	17.52
Pro	0.90	1.17	0.88	0.88	0.93	1.05	1.05	1.07	1.20	1.04	1.02	0.12	11.34

^1^ Determined by analysis and each value based on duplicate determinations. CP, crude protein; EE, ether extract; CF, crude fiber; NDF, neutral detergent fiber; ADF, acid detergent fiber; CA, crude ash; NFE, nitrogen-free extract; ST, starch; PA, phytic acid; TN, tannin; EAAs, essential amino acids; Val, valine; Met, methionine; Ile, isoleucine; Leu, leucine; Thr, threonine; Phe, phenylalanine; Lys, lysine; His, histidine; Arg, arginine; Trp, tryptophan; NEAAs, non-essential amino acids; Asp, aspartic acid; Ser, serine; Glu, glutamic acid; Gly, glycine; Ala, alanine; Cys, cysteine; Tyr, tyrosine; Pro, proline; SD, standard deviation; CV, coefficient of variation.

**Table 3:** Composition and nutrient levels of 1 NFD and 10 tested sorghum diets for chickens (as-fed basis).

Item	NFD	Tested Sorghum Diets									
		A1	A2	A3	A4	A5	A6	A7	A8	A9	A10
Ingredients, %											
Sorghum	-	95.66	95.66	95.66	95.66	95.66	95.66	95.66	95.66	95.66	95.66
Corn starch	63.81	-	-	-	-	-	-	-	-	-	-
Glucose ^1^	27.00	-	-	-	-	-	-	-	-	-	-
CaHPO_4_ ^1^	1.91	1.80	1.80	1.80	1.80	1.80	1.80	1.80	1.80	1.80	1.80
Limestone ^1^	1.16	1.30	1.30	1.30	1.30	1.30	1.30	1.30	1.30	1.30	1.30
NaCl ^1^	0.18	0.30	0.30	0.30	0.30	0.30	0.30	0.30	0.30	0.30	0.30
Cellulose ^1^	5.00	-	-	-	-	-	-	-	-	-	-
Choline chloride ^1^ (50%)	0.15	0.15	0.15	0.15	0.15	0.15	0.15	0.15	0.15	0.15	0.15
Sodium bicarbonate ^1^	0.15	0.15	0.15	0.15	0.15	0.15	0.15	0.15	0.15	0.15	0.15
Micronutrient premix ^1,2^	0.23	0.23	0.23	0.23	0.23	0.23	0.23	0.23	0.23	0.23	0.23
Antioxidant	0.01	0.01	0.01	0.01	0.01	0.01	0.01	0.01	0.01	0.01	0.01
TiO_2_	0.40	0.40	0.40	0.40	0.40	0.40	0.40	0.40	0.40	0.40	0.40
Total	100.00	100.00	100.00	100.00	100.00	100.00	100.00	100.00	100.00	100.00	100.00
Nutrient levels											
ME ^3^, kcal/kg	2848	2812	2812	2812	2812	2812	2812	2812	2812	2812	2812
CP ^4^, %	0.19	7.55	9.58	7.53	7.93	8.25	9.07	8.66	9.65	9.60	9.74
Lys ^4^, %	-	0.20	0.20	0.20	0.20	0.20	0.20	0.20	0.20	0.20	0.20
Met ^4^, %	-	0.14	0.14	0.14	0.14	0.14	0.14	0.14	0.14	0.14	0.14
Thr ^4^, %	-	0.27	0.27	0.27	0.27	0.27	0.27	0.27	0.27	0.27	0.27
Trp ^4^, %	-	0.09	0.09	0.09	0.09	0.09	0.09	0.09	0.09	0.09	0.09
Met + Cys ^4^, %	-	0.29	0.29	0.29	0.29	0.29	0.29	0.29	0.29	0.29	0.29
Ca ^3^, %	0.89	1.04	1.04	1.04	1.04	1.04	1.04	1.04	1.04	1.04	1.04
TP ^3^, %	0.38	0.69	0.69	0.69	0.69	0.69	0.69	0.69	0.69	0.69	0.69
Nonphytate P ^3^, %	0.37	0.43	0.43	0.43	0.43	0.43	0.43	0.43	0.43	0.43	0.43

^1^ Feed grade. ^2^ Provided per kilogram of diet: vitamin A 10,000 IU; cholecalciferol 3750 IU; vitamin E 27.5 IU; vitamin K 2.5 mg; vitamin B_1_ 2.5 mg; vitamin B_2_ 8.0 mg; vitamin B_6_ 3.75 mg; vitamin B_12_ 0.025 mg; calcium pantothenate 12.5 mg; niacin 45 mg; folate 125 mg; biotin 0.125 mg; zeolite 1347.8 mg; Cu (CuSO_4_·5H_2_O), 7 mg; Fe (FeSO_4_·H_2_O) 80 mg; Zn (ZnSO_4_·H_2_O) 55 mg; Mn (MnSO_4_·H_2_O) 60 mg; Se (Na_2_SeO_3_) 0.15 mg; I (Ca(IO_3_)_2_·H_2_O) 0.5 mg. ^3^ Calculated values. ^4^ Measured values based on duplicate determinations. NFD, nitrogen-free diet; TiO_2_, titanium dioxide; ME, metabolizable energy; CP, crude protein; Lys, lysine; Met, methionine; Thr, threonine; Trp, tryptophan; Cys, cysteine; Ca, calcium; TP, total phosphorus.

**Table 4:** AIAADs of sorghums from different sources for chickens ^1^.

Item (%)	Sorghum Sources										Mean	Pooled SE	CV (%)	*p*-Value
	A1	A2	A3	A4	A5	A6	A7	A8	A9	A10				
EAAs														
Val	39.6 ^c^	54.7 ^a^	17.8 ^d^	36.9 ^c^	34.5 ^c^	50.9 ^ab^	18.6 ^d^	34.5 ^c^	50.4 ^ab^	42.7 ^bc^	38.1	3.3	33.2	<0.001
Met	39.6 ^b^	68.9 ^a^	23.4 ^d^	40.8 ^b^	35.6 ^bc^	65.9 ^a^	24.8 ^cd^	43.1 ^b^	62.7 ^a^	58.0 ^a^	46.3	4.2	35.9	<0.001
Ile	40.1 ^b^	64.7 ^a^	19.6 ^c^	38.4 ^b^	31.6 ^b^	61.7 ^a^	16.1 ^c^	36.6 ^b^	58.2 ^a^	54.7 ^a^	42.2	3.5	40.7	<0.001
Leu	44.1 ^c^	72.9 ^a^	27.8 ^fg^	37.2 ^de^	32.6 ^ef^	75.2 ^a^	23.6 ^g^	39.7 ^cd^	71.0 ^a^	63.3 ^b^	48.7	2.2	40.8	<0.001
Thr	32.2 ^abcd^	43.0 ^a^	17.4 ^e^	32.6 ^abc^	26.8 ^cde^	37.2 ^abc^	20.7 ^de^	27.8 ^bcde^	39.4 ^ab^	31.7 ^abcd^	30.9	4.1	25.9	0.001
Phe	45.1 ^c^	69.1 ^ab^	28.8 ^ef^	39.0 ^cd^	35.6 ^de^	70.9 ^a^	25.7 ^f^	45.4 ^c^	70.5 ^ab^	63.2 ^b^	49.3	2.6	35.8	<0.001
Lys	39.2	51.4	32.1	51.2	50.9	49.1	34.2	43.1	53.7	41.5	44.6	6.5	17.3	0.232
His	38.1 ^cd^	59.5 ^a^	21.9 ^e^	42.5 ^bc^	38.9 ^cd^	54.1 ^a^	23.2 ^e^	31.0 ^de^	53.1 ^a^	51.7 ^ab^	41.4	3.3	31.9	<0.001
Arg	50.7 ^b^	67.6 ^a^	29.8 ^c^	49.6 ^b^	46.1 ^b^	69.2 ^a^	36.1 ^c^	50.1 ^b^	71.3 ^a^	67.0 ^a^	53.8	3.5	27.0	<0.001
Trp	41.6 ^b^	58.7 ^a^	24.3 ^d^	36.7 ^bc^	32.2 ^bcd^	59.2 ^a^	29.4 ^cd^	37.8 ^bc^	62.6 ^a^	53.4 ^a^	43.6	3.7	31.7	<0.001
NEAAs														
Asp	43.3 ^d^	63.3 ^a^	25.3 ^e^	43.2 ^d^	39.0 ^d^	58.3 ^ab^	34.8 ^d^	44.2 ^cd^	58.5 ^ab^	53.0 ^bc^	46.3	3.3	25.7	<0.001
Ser	39.9 ^c^	56.4 ^a^	18.0 ^f^	35.6 ^cde^	28.2 ^de^	53.9 ^ab^	27.0 ^ef^	37.9 ^cd^	53.1 ^ab^	45.0 ^bc^	39.5	3.4	32.4	<0.001
Glu	41.1 ^c^	72.6 ^a^	25.3 ^d^	39.6 ^c^	37.4 ^c^	73.6 ^a^	28.7 ^d^	44.3 ^c^	68.9 ^ab^	65.4 ^b^	49.7	2.4	37.4	<0.001
Gly	46.1 ^bc^	58.6 ^a^	27.8 ^e^	45.4 ^bc^	39.3 ^cd^	47.5 ^bc^	28.4 ^e^	35.6 ^de^	52.0 ^ab^	46.2 ^bc^	42.7	3.4	23.2	<0.001
Ala	47.8 ^c^	73.3 ^a^	29.1 ^e^	42.3 ^cd^	39.7 ^d^	75.5 ^a^	29.2 ^e^	45.8 ^cd^	73.1 ^ab^	66.9 ^b^	52.3	2.2	35.0	<0.001
Cys	24.3 ^c^	59.0 ^a^	12.4 ^de^	42.4 ^a^	36.8 ^b^	39.2 ^b^	8.5 ^e^	21.1 ^cd^	38.9 ^b^	37.2 ^b^	32.0	3.7	47.8	<0.001
Tyr	36.6 ^c^	67.3 ^ab^	18.5 ^de^	32.6 ^c^	23.9 ^d^	74.2 ^a^	12.5 ^e^	16.1 ^de^	68.8 ^ab^	66.0 ^b^	41.7	2.7	59.4	<0.001
Pro	49.4 ^cd^	62.3 ^a^	40.3 ^de^	37.7 ^e^	15.6 ^g^	59.4 ^ab^	23.1 ^fg^	30.9 ^ef^	52.6 ^bc^	53.6 ^abc^	42.5	3.2	36.9	<0.001

^1^ Values represent the means of 6 replicate cages (4 birds per replicate cage) (*n* = 6); Different lowercase letters within the same row indicate significant differences (*p* < 0.05). AIAADs, apparent ileal amino acid digestibilities; EAAs, essential amino acids; Val, valine; Met, methionine; Ile, isoleucine; Leu, leucine; Thr, threonine; Phe, phenylalanine; Lys, lysine; His, histidine; Arg, arginine; Trp, tryptophan; NEAAs, non-essential amino acids; Asp, aspartic acid; Ser, serine; Glu, glutamic acid; Gly, glycine; Ala, alanine; Cys, cysteine; Tyr, tyrosine; Pro, proline; SE, standard error; CV, coefficient of variation.

**Table 5:** SIAADs of sorghums from different sources for chickens ^1^.

Item (%)	Sorghum Sources										Mean	Pooled SE	CV (%)	*p*-Value	BEL (%)
	A1	A2	A3	A4	A5	A6	A7	A8	A9	A10					
EAAs															
Val	59.5 ^bcd^	70.5 ^a^	38.5 ^e^	55.4 ^cd^	53.2 ^cd^	70.4 ^a^	36.3 ^e^	52.0 ^d^	68.6 ^ab^	62.1 ^abc^	56.7	3.3	21.5	<0.001	0.08
Met	49.5 ^b^	76.6 ^a^	33.4 ^cd^	50.4 ^b^	45.2 ^bc^	75.7 ^a^	32.8 ^d^	50.9 ^b^	71.4 ^a^	67.9 ^a^	55.4	4.2	29.8	<0.001	0.01
Ile	55.0 ^b^	76.4 ^a^	34.2 ^d^	51.8 ^bc^	45.2 ^c^	75.4 ^a^	28.6 ^d^	48.9 ^bc^	71.3 ^a^	68.3 ^a^	55.5	3.5	30.5	<0.001	0.04
Leu	52.0 ^c^	79.0 ^a^	35.2 ^ef^	44.4 ^d^	39.7 ^de^	81.9 ^a^	29.9 ^f^	45.9 ^cd^	77.5 ^a^	69.9 ^b^	55.5	2.2	35.4	<0.001	0.07
Thr	59.5 ^a^	64.3 ^a^	45.4 ^cd^	57.6 ^ab^	52.6 ^abcd^	62.6 ^a^	42.4 ^d^	50.1 ^bcd^	63.5 ^a^	57.0 ^abc^	55.5	4.1	13.8	0.002	0.07
Phe	57.3 ^c^	78.5 ^ab^	40.6 ^ef^	50.0 ^cd^	46.6 ^de^	81.2 ^a^	35.6 ^f^	55.2 ^c^	80.4 ^ab^	73.3 ^b^	59.9	2.6	28.7	<0.001	0.04
Lys	66.3	70.5	56.8	74.1	75.0	72.3	54.6	64.2	75.1	64.0	67.3	6.5	11.0	0.287	0.06
His	49.1 ^bc^	67.7 ^a^	33.6 ^d^	53.0 ^b^	48.9 ^bc^	65.7 ^a^	33.0 ^d^	40.8 ^cd^	63.1 ^a^	63.2 ^a^	51.8	3.3	25.1	<0.001	0.02
Arg	66.5 ^b^	80.7 ^a^	47.1 ^c^	64.9 ^b^	62.0 ^b^	82.7 ^a^	50.1 ^c^	64.4 ^b^	83.7 ^a^	80.1 ^a^	68.2	3.5	19.5	<0.001	0.05
Trp	58.7 ^bc^	71.3 ^a^	40.1 ^e^	52.4 ^cd^	48.4 ^cde^	73.5 ^a^	42.0 ^de^	51.5 ^cd^	75.8 ^a^	67.1 ^ab^	58.1	3.7	22.7	<0.001	0.01
NEAAs															
Asp	61.1 ^bc^	76.8 ^a^	43.1 ^e^	58.7 ^c^	55.2 ^cd^	74.1 ^a^	48.1 ^de^	58.7 ^bc^	73.7 ^a^	68.6 ^ab^	61.8	3.3	18.4	<0.001	0.09
Ser	61.3 ^b^	73.6 ^a^	40.3 ^d^	55.6 ^bc^	49.4 ^cd^	74.0 ^a^	44.5 ^d^	55.7 ^bc^	72.0 ^a^	64.9 ^ab^	59.1	3.4	20.5	<0.001	0.07
Glu	50.3 ^c^	79.1 ^ab^	34.1 ^d^	47.6 ^c^	45.2 ^c^	81.0 ^a^	35.5 ^d^	51.1 ^c^	76.1 ^ab^	72.7 ^b^	57.3	2.4	31.7	<0.001	0.12
Gly	63.0 ^ab^	72.5 ^a^	46.9 ^d^	62.7 ^ab^	57.2 ^bc^	65.8 ^ab^	44.5 ^d^	51.5 ^cd^	68.3 ^a^	64.0 ^ab^	59.6	3.4	15.6	<0.001	0.05
Ala	55.4 ^c^	79.0 ^ab^	36.5 ^e^	49.0 ^cd^	46.5 ^d^	82.0 ^a^	35.4 ^e^	51.9 ^cd^	79.4 ^ab^	73.3 ^b^	58.8	2.2	30.7	<0.001	0.05
Cys	47.3 ^cd^	73.6 ^a^	36.1 ^e^	60.8 ^b^	54.6 ^bc^	63.0 ^ab^	29.5 ^e^	39.9 ^de^	59.6 ^b^	59.9 ^b^	52.4	3.7	26.3	<0.001	0.03
Tyr	45.1 ^c^	74.2 ^ab^	26.8 ^de^	40.3 ^c^	31.7 ^d^	80.7 ^a^	19.1 ^e^	24.5 ^de^	75.0 ^ab^	72.4 ^b^	49.0	2.9	49.3	<0.001	0.02
Pro	58.9 ^cd^	72.0 ^a^	49.5 ^de^	48.4 ^e^	26.2 ^g^	69.0 ^ab^	30.7 ^fg^	38.4 ^f^	61.8 ^bc^	63.4 ^abc^	51.8	3.2	30.7	<0.001	0.06

^1^ Values represent the means of 6 replicate cages (4 birds per replicate cage) (*n* = 6); Different lowercase letters within the same row indicate significant differences (*p* < 0.05). SIAADs, standardized ileal amino acid digestibilities; EAAs, essential amino acids; Val, valine; Met, methionine; Ile, isoleucine; Leu, leucine; Thr, threonine; Phe, phenylalanine; Lys, lysine; His, histidine; Arg, arginine; Trp, tryptophan; NEAAs, non-essential amino acids; Asp, aspartic acid; Ser, serine; Glu, glutamic acid; Gly, glycine; Ala, alanine; Cys, cysteine; Tyr, tyrosine; Pro, proline; SE, standard error; CV, coefficient of variation.

**Table 6:** Correlations between chemical compositions (air-dry basis) and SIAADs of sorghums for chickens.

Item (%)	CP (%)	EE (%)	CA (%)	CF (%)	ADF (%)	NDF (%)	PA (%)	TN (%)	NFE (%)	ST (%)
EAAs										
Val	0.361	−0.042	−0.293	0.237	0.317	0.424	−0.369	−0.772 *	0.291	0.027
Met	0.576	−0.043	−0.121	0.113	0.426	0.507	−0.229	−0.891 **	0.143	0.149
Ile	0.466	−0.074	−0.183	0.170	0.404	0.468	−0.338	−0.872 **	0.189	0.035
Leu	0.546	−0.089	−0.053	0.174	0.492	0.525	−0.174	−0.942 **	0.131	0.130
Thr	0.194	−0.009	−0.414	0.223	0.204	0.291	−0.440	−0.682 *	0.362	−0.132
Phe	0.562	−0.119	−0.027	0.157	0.545	0.578	−0.193	−0.943 **	0.137	0.125
Lys	0.124	0.297	−0.509	0.254	−0.092	0.231	−0.315	−0.239	0.158	−0.059
His	0.482	0.171	−0.212	0.189	0.272	0.438	−0.356	−0.765 *	0.078	0.039
Arg	0.585	0.038	−0.050	0.109	0.498	0.609	−0.277	−0.848 **	0.142	0.117
Trp	0.533	−0.015	−0.045	0.137	0.516	0.574	−0.200	−0.887 **	0.203	0.111
NEAAs										
Asp	0.522	−0.029	−0.165	0.109	0.396	0.478	−0.231	−0.839 **	0.276	0.172
Ser	0.453	−0.096	−0.170	0.090	0.430	0.459	−0.262	−0.845 **	0.353	0.100
Glu	0.633	−0.032	−0.020	0.128	0.492	0.565	−0.156	−0.924 **	0.096	0.209
Gly	0.258	0.071	−0.397	0.229	0.165	0.285	−0.438	−0.690 *	0.281	−0.085
Ala	0.584	−0.055	−0.032	0.160	0.513	0.573	−0.186	−0.926 **	0.129	0.159
Cys	0.312	0.198	−0.456	0.224	−0.014	0.197	−0.413	−0.596	0.079	−0.036
Tyr	0.527	0.088	−0.022	0.190	0.445	0.517	−0.239	−0.885 **	0.009	0.034
Pro	0.183	−0.275	−0.153	0.080	0.370	0.200	−0.376	−0.825 **	0.216	−0.260

* represents a significant difference (*p* < 0.05); ** represents a highly significant difference (*p* < 0.01). The SIAAD values of 9 tested sorghum (*n* = 9) were used for correlation (r) analysis.

**Table 7:** Correlations between EAAs (air-dry basis) and SIAADs of sorghums for chickens.

Items (%)	Val (%)	Met (%)	Ile (%)	Leu (%)	Thr (%)	Phe (%)	Lys (%)	His (%)	Arg (%)	Trp (%)
EAAs										
Val	0.282	−0.486	0.357	0.440	0.154	0.319	−0.253	0.367	0.265	0.381
Met	0.460	−0.360	0.533	0.640	0.331	0.517	−0.025	0.427	0.428	0.400
Ile	0.331	−0.360	0.408	0.518	0.195	0.386	−0.177	0.322	0.346	0.399
Leu	0.413	−0.239	0.478	0.593	0.271	0.452	−0.074	0.319	0.470	0.459
Thr	0.165	−0.458	0.227	0.296	0.025	0.168	−0.366	0.270	0.141	0.315
Phe	0.406	−0.270	0.478	0.589	0.269	0.457	−0.081	0.326	0.507	0.474
Lys	0.053	−0.572	0.092	0.115	−0.016	0.065	−0.299	0.510	0.040	−0.171
His	0.296	−0.439	0.359	0.477	0.158	0.330	−0.198	0.364	0.280	0.254
Arg	0.407	−0.472	0.477	0.575	0.271	0.445	−0.103	0.393	0.477	0.438
Trp	0.415	−0.380	0.473	0.566	0.267	0.424	−0.106	0.337	0.494	0.510
NEAAs										
Asp	0.493	−0.485	0.559	0.639	0.364	0.519	−0.031	0.457	0.393	0.489
Ser	0.453	−0.463	0.517	0.587	0.315	0.464	−0.085	0.375	0.383	0.554
Glu	0.491	−0.322	0.559	0.672 *	0.360	0.541	0.012	0.411	0.500	0.450
Gly	0.195	−0.465	0.257	0.344	0.056	0.206	−0.328	0.297	0.124	0.278
Ala	0.434	−0.319	0.502	0.613	0.296	0.476	−0.062	0.361	0.495	0.471
Cys	0.195	−0.404	0.257	0.366	0.083	0.247	−0.233	0.405	0.038	0.005
Tyr	0.292	−0.220	0.346	0.482	0.135	0.311	−0.180	0.210	0.424	0.364
Pro	0.100	0.061	0.159	0.273	−0.050	0.125	−0.295	−0.133	0.182	0.426

* represents a significant difference (*p* < 0.05). The SIAAD values of 9 tested sorghum (*n* = 9) were used for correlation (r) analysis. EAAs, essential amino acids; SIAADs, standardized ileal amino acid digestibilities; Val, valine; Met, methionine; Ile, isoleucine; Leu, leucine; Thr, threonine; Phe, phenylalanine; Lys, lysine; His, histidine; Arg, arginine; Trp, tryptophan; NEAAs, non-essential amino acids; Asp, aspartic acid; Ser, serine; Glu, glutamic acid; Gly, glycine; Ala, alanine; Cys, cysteine; Tyr, tyrosine; Pro, proline.

**Table 8:** Correlations between NEAAs (air-dry basis) and SIAADs of sorghums for chickens.

Items (%)	Asp (%)	Ser (%)	Glu (%)	Gly (%)	Ala (%)	Cys (%)	Tyr (%)	Pro (%)
EAAs								
Val	0.054	0.288	0.373	0.244	0.395	−0.308	0.641	0.523
Met	0.237	0.460	0.576	0.376	0.591	−0.184	0.541	0.690 *
Ile	0.098	0.343	0.447	0.270	0.467	−0.186	0.524	0.589
Leu	0.168	0.398	0.524	0.354	0.545	−0.054	0.481	0.698 *
Thr	−0.058	0.162	0.221	0.173	0.274	−0.289	0.666 *	0.397
Phe	0.156	0.382	0.521	0.327	0.528	−0.087	0.481	0.698 *
Lys	−0.074	0.037	0.080	−0.004	0.109	−0.392	0.760 *	0.159
His	0.088	0.292	0.407	0.234	0.454	−0.248	0.550	0.511
Arg	0.171	0.347	0.508	0.309	0.523	−0.286	0.555	0.647
Trp	0.163	0.340	0.493	0.359	0.523	−0.191	0.565	0.686 *
NEAAs								
Asp	0.271	0.466	0.575	0.441	0.601	−0.317	0.635	0.696 *
Ser	0.218	0.411	0.517	0.421	0.548	−0.306	0.624	0.672 *
Glu	0.261	0.471	0.609	0.404	0.622	−0.140	0.499	0.738 *
Gly	−0.014	0.209	0.270	0.197	0.330	−0.291	0.634	0.408
Ala	0.191	0.404	0.545	0.358	0.560	−0.132	0.506	0.707 *
Cys	0.041	0.276	0.307	0.157	0.362	−0.231	0.580	0.345
Tyr	0.053	0.253	0.404	0.239	0.453	−0.023	0.407	0.582
Pro	−0.113	0.141	0.190	0.136	0.243	0.160	0.187	0.385

* represents a significant difference (*p* < 0.05). The SIAAD values of 9 tested sorghums (*n* = 9) were used for correlation (r) analysis. NEAAs, non-essential amino acids; SIAADs, standardized ileal amino acid digestibilities; EAAs, essential amino acids; Val, valine; Met, methionine; Ile, isoleucine; Leu, leucine; Thr, threonine; Phe, phenylalanine; Lys, lysine; His, histidine; Arg, arginine; Trp, tryptophan; Asp, aspartic acid; Ser, serine; Glu, glutamic acid; Gly, glycine; Ala, alanine; Cys, cysteine; Tyr, tyrosine; Pro, proline. SIAADs, standardized ileal amino acid digestibilities; CP, crude protein; EE, ether extract; CA, crude ash; CF, crude fiber; ADF, acid detergent fiber; NDF, neutral detergent fiber; PA, phytic acid; TN, tannin; NFE, nitrogen-free extract; ST, starch; EAAs, essential amino acids; Val, valine; Met, methionine; Ile, isoleucine; Leu, leucine; Thr, threonine; Phe, phenylalanine; Lys, lysine; His, histidine; Arg, arginine; Trp, tryptophan; NEAAs, non-essential amino acids; Asp, aspartic acid; Ser, serine; Glu, glutamic acid; Gly, glycine; Ala, alanine; Cys, cysteine; Tyr, tyrosine; Pro, proline.

**Table 9:** Established predicting models of SIAADs in sorghums for chickens based on their chemical compositions and amino acid profiles (air-dry basis)^1^.

Predicting Models	*R* ^2^	RMSE	*p*-Value
EAAs (%)			
SID Val = 169.01 − 34.46 CA − 19.02 TN − 58.60 Ser − 289.55 Met	0.981	0.05	0.001
SID Met = 142.97 − 28.38 TN − 396.75 Met − 406.55 Trp	0.978	1.01	<0.001
SID Ile = 177.77 − 34.95 CA − 27.56 TN − 88.13 Gly − 316.31 Met	0.983	0.01	<0.001
SID Leu = 74.07 − 34.31 PA − 27.48 TN + 99.53 Tyr	0.981	0.04	<0.001
SID Thr = 111.53 − 32.04 CA − 10.19 TN	0.748	3.21	0.016
SID Phe = 121.08 − 16.98 CA − 27.42 TN − 218.49 Met	0.981	0.12	<0.001
SID Lys = 62.42 − 344.44 Gly + 68.01 Ala + 168.72 Tyr	0.988	0.01	<0.001
SID His = 129.22 + 6.13 EE − 26.61 TN − 311 Met − 31.04 Arg − 321.57 Trp	0.988	0.07	0.004
SID Arg = 141.10 − 22.07 TN − 397.16 Met − 297.30 Trp	0.986	0.01	<0.001
SID Trp = 91.05 − 19.54 TN − 262.92 Met	0.934	3.78	<0.001
NEAAs (%)			
SID Asp = 121.65 − 18.66 CA − 16.90 TN − 267.27 Met	0.986	0.02	<0.001
SID Ser = 121.74 − 20.48 CA − 17.71 TN − 262.48 Met	0.980	2.10	<0.001
SID Glu = 136.83 − 30.60 TN − 370.93 Met − 326.42 Trp	0.988	0.01	<0.001
SID Gly = 116.78 + 7.14 EE − 45.93 CA − 15.02 TN	0.898	0.05	0.007
SID Ala = 132.31 − 30.13 TN − 356.73 Met − 276.11 Trp	0.983	0.10	<0.001
SID Cys = 150.76 + 13.49 EE − 67.37 CA − 32.09 PA − 20.31 TN	0.968	3.58	0.003
SID Tyr = 29.04 + 19.10 EE − 38.44 TN	0.898	0.03	0.001
SID Pro = 356.87 − 1.50 NDF − 30.11 TN − 3.98 ST	0.944	4.71	0.002

^1^ The SIAAD values of 9 tested sorghums (*n* = 9) were used to construct the SIAAD-predicting models. SIAADs, standardized ileal amino acid digestibilities; SID, standardized ileal digestibility; CA, crude ash (%); TN, tannin (%); PA, phytic acid (%); EE, ether extract (%); NDF, neutral detergent fiber (%); ST, starch (%); EAAs, essential amino acids; Val, valine; Met, methionine; Ile, isoleucine; Leu, leucine; Thr, threonine; Phe, phenylalanine; Lys, lysine; His, histidine; Arg, arginine; Trp, tryptophan; NEAAs, non-essential amino acids; Asp, aspartic acid; Ser, serine; Glu, glutamic acid; Gly, glycine; Ala, alanine; Cys, cysteine; Tyr, tyrosine; Pro, proline; *R*^2^, coefficient of determination; RMSE, root mean square error.

**Table 10:** Verification of the accuracy of predicting models for SIAADs in sorghums for chickens.

Item (%)	Determined Value of SIAAD in Sorghum A6 ^1^ (%)	Predicted Value of SIAAD in Sorghum A6 (%)	Difference Value ^2^ (%)	Accuracy Evaluation
SID Val	70.4 ± 13.1	70.1	0.3	Accurate
SID Met	75.7 ± 14.0	76.7	1.0	Accurate
SID Ile	75.4 ± 13.1	76.7	1.3	Accurate
SID Leu	81.9 ± 9.2	68.8	13.1	Inaccurate
SID Thr	62.6 ± 14.9	62.6	0.0	Accurate
SID Phe	81.2 ± 10.0	79.1	2.1	Accurate
SID Lys	72.3 ± 21.5	70.3	2.0	Accurate
SID His	65.7 ± 10.3	65.7	0.0	Accurate
SID Arg	82.7 ± 11.7	85.3	2.6	Accurate
SID Trp	73.5 ± 13.4	72.1	1.4	Accurate
SID Asp	74.1 ± 12.1	74.3	0.2	Accurate
SID Ser	74.0 ± 12.1	71.9	2.1	Accurate
SID Glu	81.0 ± 9.4	79.7	1.3	Accurate
SID Gly	65.8 ± 13.2	61.1	4.7	Accurate
SID Ala	82.0 ± 8.6	80.8	1.2	Accurate
SID Cys	63.0 ± 16.5	47.7	15.3	Accurate
SID Tyr	80.7 ± 10.5	65.7	15.0	Inaccurate
SID Pro	69.0 ± 11.5	67.4	1.6	Accurate

^1^ The determined value is the mean ± standard deviation (SD) of 6 replicate values (*n* = 6). ^2^ The difference value equals the deviation between determined and predicted values. SIAADs, standardized ileal amino acid digestibilities; SID, standardized ileal digestibility; Val, valine; Met, methionine; Ile, isoleucine; Leu, leucine; Thr, threonine; Phe, phenylalanine; Lys, lysine; His, histidine; Arg, arginine; Trp, tryptophan; Asp, aspartic acid; Ser, serine; Glu, glutamic acid; Gly, glycine; Ala, alanine; Cys, cysteine; Tyr, tyrosine; Pro, proline.

## Data Availability

The original contributions presented in this study are included in the article. Further inquiries can be directed to the corresponding author.

## References

[1] Wang M., Toghyani M., Macelline S.P., Lemme A., Holmes A.J., Selle P.H., Liu S.Y. (2025). Sorghum surpasses wheat as a feed grain for broiler chickens following dietary crude protein reductions. J. Anim. Sci. Biotechnol..

[2] Macelline S.P., Godwin I.D., Liu G., Restall J., Cantor D.I., McInerney B.V., Toghyani M., Chrystal P.V., Selle P.H., Liu S.Y. (2024). Transgenic, high-protein sorghums display promise in poultry diets in an initial comparison. Poult. Sci..

[3] Sasia S., Bridges W., Arguelles-Ramos M. (2025). Determination of the standardized ileal amino acid digestibility of U.S. tannin-free sorghum in broilers. Agriculture.

[4] Duodu K.G., Taylor J.N., Belton P.S., Hamaker B.R. (2003). Factors affecting sorghum protein digestibility. J. Cereal Sci..

[5] Selle P.H., Cadogan D.J., Li X., Bryden W.L. (2010). Implications of sorghum in broiler chicken nutrition. Anim. Feed. Sci. Technol..

[6] Pan L., Feng S.X., Wang L., Zhu W.Y. (2022). Comparative digestion and fermentation characteristics of low-tannin or high-tannin sorghum grain in the porcine gastrointestinal tract. J. Anim. Sci..

[7] Keyata E.O., Tola Y.B., Bultosa G., Forsido S.F. (2021). Premilling treatments effects on nutritional composition, antinutritional factors, and in vitro mineral bioavailability of the improved Assosa I sorghum variety (*Sorghum bicolor* L.). Food Sci. Nutr..

[8] Hodges H.E., Walker H.J., Cowieson A.J., Falconer R.J., Cameron D.D. (2021). Latent anti-nutrients and unintentional breeding consequences in australian sorghum bicolor varieties. Front. Plant Sci..

[9] Zhang G., Zhang G., Zhao J., Liu L., Zhang Z. (2024). Effect of extrusion on available energy and amino acid digestibility of barley, wheat, sorghum, and broken rice in growing pigs. Anim. Biosci..

[10] Barua M., Abdollahi M.R., Zaefarian F., Wester T.J., Girish C.K., Chrystal P.V., Ravindran V. (2021). An investigation into the influence of age on the standardized amino acid digestibility of wheat and sorghum in broilers. Poult. Sci..

[11] Feng G.Y., Li R., Jiang X.J., Yang G., Tian M.Z., Xiang Q., Liu X.J., Ouyang Q., Long C.M., Huang R.L. (2023). Prediction of available energy and amino acid digestibility of Chinese sorghum fed to growing-finishing pigs. J. Anim. Sci..

[12] Sheikhhasan B.S., Moravej H., Ghaziani F., Esteve-Garcia E., Kim W.K. (2020). Relationship between chemical composition and standardized ileal digestible amino acid contents of corn grain in broiler chickens. Poult. Sci..

[13] Sheikhhasan B.S., Moravej H., Shivazad M., Ghaziani F., Esteve-Garcia E., Kim W.K. (2020). Prediction of the total and standardized ileal digestible amino acid contents from the chemical composition of soybean meals of different origin in broilers. Poult. Sci..

[14] Yun X.L., Liu X.B., Cheng Z.C., Ji Y.R., Guo Y.M., Yuan J.M., Nie W. (2023). Determination and prediction of standardized ileal amino acid digestibility of wheat in broilers. Poult. Sci..

[15] (2020). Nutritional Requirements of Yellow Feather Broilers.

[16] Ravindran V., Hew L., Ravindran G., Bryden W.L. (2005). Apparent ileal digestibility of amino acids in dietary ingredients for broiler chickens. Anim. Sci..

[17] Luzardo-Ocampo I., Ramírez-Jiménez A.K., Cabrera-Ramírez H., Rodríguez-Castillo N., Campos-Vega R., Loarca-Piña G., Gaytán-Martínez M. (2020). Impact of cooking and nixtamalization on the bioaccessibility and antioxidant capacity of phenolic compounds from two sorghum varieties. Food Chem..

[18] AOAC (Association of Official Analytical Chemists) (2016). Official Methods of Analysis.

[19] Titgemeyer E.C., Armendariz C.K., Bindel D.J., Greenwood R.H., Löest C.A. (2001). Evaluation of titanium dioxide as a digestibility marker for cattle. J. Anim. Sci..

[20] Short F.J., Gorton P., Wiseman J., Boorman K.N. (1996). Determination of titanium dioxide added as an inert marker in chicken digestibility studies. Anim. Feed. Sci. Technol..

[21] Cao S.M., Li T.T., Shao Y.X., Zhao Y.Z., Zhang L.Y., Lu L., Zhang R.J., Hou S.S., Liao X.D., Luo X.G. (2023). Establishment and evaluation of the primary cultured tibial osteoblast model of broiler chicks. J. Integr. Agric..

[22] Du X., Chen W.P., Cao C.Y., Zhang L.Y., Wang S.C., Cui X.Y., Li T.T., Hu Y., Luo X.G. (2026). Determination of standardized mineral availability in different corn sources for broilers and establishment of prediction models. Anim. Nutr..

[23] Hu Y., Du X., Chen W.P., Zhang L.Y., Cui X.Y., Li T.T., Wang S.C., Luo X.G. (2026). Determination and prediction of standardized mineral retentions in different sources of rapeseed meals for broilers. Poult. Sci..

[24] An S.H., Sung J.Y., Kang H.K., Kong C. (2020). Additivity of ileal amino acid digestibility in diets containing corn, soybean meal, and corn distillers dried grains with solubles for male broilers. Animals.

[25] Ravindran V. (2021). Progress in ileal endogenous amino acid flow research in poultry. J. Anim. Sci. Biotechnol..

[26] Thomas L.L., Espinosa C.D., Goodband R.D., Stein H.H., Tokach M.D., Dritz S.S., Woodworth J.C., DeRouchey J.M. (2020). Nutritional evaluation of different varieties of sorghum and the effects on nursery pig growth performance. J. Anim. Sci..

[27] Olukosi O.A., Pilevar M., Ajao A.M., Veluri S., Lin Y. (2023). Determination of standardised ileal digestibility of amino acids in high-fibre feedstuffs and additivity of apparent and standardised ileal amino acids digestibility of diets containing mixtures of maize, sorghum, and soybean meal. Br. Poult. Sci..

[28] Barua M., Abdollahi M.R., Zaefarian F., Wester T.J., Girish C.K., Ravindran V. (2021). Influence of feed form on the standardised ileal amino acid digestibility of common grains for broiler chickens. Anim. Feed. Sci. Technol..

[29] Pan L., Li W., Gu X.M., Zhu W.Y. (2022). Comparative ileal digestibility of gross energy and amino acids in low and high tannin sorghum fed to growing pigs. Anim. Feed. Sci. Technol..

[30] Hagerman A.E., Butler L.G. (1981). The specificity of proanthocyanidin-protein interactions. J. Biol. Chem..

